# Facilitators, barriers, and key influencers of breastfeeding among low birthweight infants: a qualitative study in India, Malawi, and Tanzania

**DOI:** 10.1186/s13006-023-00597-7

**Published:** 2023-11-08

**Authors:** Linda Vesel, Emily Benotti, Sarah Somji, Roopa M Bellad, Umesh Charantimath, Sangappa M Dhaded, Shivaprasad S Goudar, Chandrashekhar Karadiguddi, Geetanjali Mungarwadi, Sunil S Vernekar, Rodrick Kisenge, Karim Manji, Nahya Salim, Abraham Samma, Christopher R Sudfeld, Irving F Hoffman, Tisungane Mvalo, Melda Phiri, Friday Saidi, Jennifer Tseka, Mercy Tsidya, Bethany A Caruso, Christopher P Duggan, Kiersten Israel-Ballard, Anne CC Lee, Kimberly L Mansen, Stephanie L Martin, Krysten North, Melissa F Young, Eliza Fishman, Katelyn Fleming, Katherine EA Semrau, Lauren Spigel, Danielle E Tuller, Natalie Henrich

**Affiliations:** 1 Ariadne Labs at Brigham and Women’s Hospital, Harvard T.H. Chan School of Public Health, Boston, MA USA; 2 Department of Pediatrics and Child Health, Muhimbili University of Health and Allied Sciences, Dar es Salaam, Tanzania; 3 KLE Academy of Higher Education and Research’s Jawaharlal Nehru Medical College, Belgaum, Karnataka India; 4 Departments of Global Health and Population and Nutrition, Harvard T.H. Chan School of Public Health, Boston, MA USA; 5 Institute for Global Health and Infectious Diseases, University of North Carolina School of Medicine, Chapel Hill, NC USA; 6 University of North Carolina Project Malawi, Lilongwe, Malawi; 7 Department of Pediatrics, School of Medicine, University of North Carolina at Chapel Hill, Chapel Hill, NC USA; 8 Department of Obstetrics and Gynecology, School of Medicine, University of North Carolina at Chapel Hill, Chapel Hill, NC USA; 9 Hubert Department of Global Health, Rollins School of Public Health, Emory University, Atlanta, GA USA; 10 Center for Nutrition, Boston Children’s Hospital, Boston, MA USA; 11 Maternal, Newborn, Child Health and Nutrition Program, PATH, Seattle, WA USA; 12 Department of Pediatric Newborn Medicine, Brigham and Women’s Hospital, Boston, MA USA; 13 Department of Pediatrics, Harvard Medical School, Boston, MA USA; 14 Department of Nutrition, Gillings School of Global Public Health, University of North Carolina at Chapel Hill, Chapel Hill, NC USA; 15 Department of Medicine, Harvard Medical School, Boston, MA USA; 16 Department of Maternal and Child Health, Gillings School of Global Public Health, University of North Carolina, Chapel Hill, NC USA

**Keywords:** Low birthweight, Breastfeeding, Infant feeding, Facilitators, Barriers, Qualitative

## Abstract

**Background:**

Low birthweight (LBW) infants are at increased risk of morbidity and mortality. Exclusive breastfeeding up to six months is recommended to help them thrive through infection prevention, growth improvements, and enhancements in neurodevelopment. However, limited data exist on the feeding experiences of LBW infants, their caregivers and key community influencers. The qualitative component of the Low Birthweight Infant Feeding Exploration (LIFE) study aimed to understand practices, facilitators, and barriers to optimal feeding options in the first six months for LBW infants in low-resource settings.

**Methods:**

This study was conducted in four sites in India, Malawi, and Tanzania from July 2019 to August 2020. We conducted 37 focus group discussions with mothers and family members of LBW infants and community leaders and 142 in-depth interviews with healthcare providers, government officials, and supply chain and donor human milk (DHM) experts. Data were analyzed using a framework approach.

**Results:**

All participants believed that mother’s own milk was best for LBW infants. Direct breastfeeding was predominant and feeding expressed breast milk and infant formula were rare. DHM was a new concept for most. Adequate maternal nutrition, lactation support, and privacy in the facility aided breastfeeding and expression, but perceived insufficient milk, limited feeding counseling, and infant immaturity were common barriers. Most believed that DHM uptake could be enabled through community awareness by overcoming misconceptions, safety concerns, and perceived family resistance.

**Conclusion:**

This study fills an evidence gap in LBW infant feeding practices and their facilitators and barriers in resource-limited settings. LBW infants face unique feeding challenges such as poor latching and tiring at the breast. Similarly, their mothers are faced with numerous difficulties, including attainment of adequate milk supply, breast pain and emotional stress. Lactation support and feeding counseling could address obstacles faced by mothers and infants by providing psychosocial, verbal and physical support to empower mothers with skills, knowledge and confidence and facilitate earlier, more and better breast milk feeding. Findings on DHM are critical to the future development of human milk banks and highlight the need to solicit partnership from stakeholders in the community and health system.

**Supplementary Information:**

The online version contains supplementary material available at 10.1186/s13006-023-00597-7.

## Background

Over the past two decades, global efforts have yielded significant progress in newborn and child survival [1, 2]. However, achievement of the Sustainable Development Goal 3.2 to end preventable deaths of newborns and children has been impeded due to a slow decline in neonatal mortality, which comprises nearly half of under-five deaths [3, 4]. More than 80% of global child deaths occur in sub-Saharan Africa and South Asia [5]. The recent *Lancet* Series on small vulnerable newborns [6] [SVNs; those born preterm, small-for-gestational age (SGA) or preterm and SGA)] has highlighted that SVNs represent more than a quarter of all global births and has elevated the urgency to address the disproportionate burden in low- and middle-income countries (LMICs). SVNs represent 99.5% of global low birthweight (LBW, < 2.5 kg) infants (born preterm and/or SGA), who account for 60–80% of neonatal deaths and face an increased risk for infection, neurodevelopmental impairment, and suboptimal growth [6–10]. Early initiation and exclusive breastfeeding (EBF) can help LBW infants thrive by preventing morbidities, improving growth, and enhancing developmental outcomes [11–15]). Prioritizing the care for SVNs, of which feeding is key, can contribute to a reduction of nearly 600,000 newborn deaths a year [11–15].

The World Health Organization (WHO) recommends EBF for all infants up to six months of age and continued breastfeeding up to two years or beyond [16]. Improvements are needed to achieve global targets given that only 44% of infants are exclusively breastfed up to six months (55% in India, 59% in Malawi and 59% in Tanzania) and less than 50% are put to the breast within the first hour of birth (42% in India, 77% in Malawi and 51% in Tanzania) [17–23]. Although global estimates for these feeding indicators are not available for LBW infants, rates are likely lower due to immaturity and other complications.

Current LBW infant feeding recommendations are based on low-quality evidence according to the Grading of Recommendations, Assessment, Development, and Evaluation (GRADE) methodology that is used to rate the quality of evidence underpinning guidelines. Most of the evidence comes from observational studies conducted in high-income settings and/or among very LBW (< 1.5 kg) infants [24]. When mother’s own milk (MOM; direct or expressed) is unavailable to LBW or preterm infants (i.e., due to separation resulting from cesarean section or other health system barriers), WHO recommends donor human milk (DHM) as the first alternative followed by human milk substitutes (i.e., infant formula). While WHO has made an effort to scale human milk banks (HMB), their acceptability and feasibility in diverse settings, particularly in resource-limited countries, has not been fully assessed. Further, it is essential to understand caregivers’ context-specific experiences of LBW infant feeding, including beliefs, practices, facilitators, and barriers [25]. Existing evidence is based on general or normal birthweight (NBW; ≥2.5 kg) infants, and little is known about facilitators and barriers of EBF provision and other feeding alternatives among LBW infants [26–28]. While some previous studies sought to understand the opinions and influence of family members and health care providers (HCPs) on LBW infant feeding practices [29–31], a gap remains in understanding the perspectives of other influencers, including community members and elders, government officials, and experts who focus on DHM and the infant feeding supply chain.

The Low Birthweight Infant Feeding Exploration (LIFE) study aims to document current feeding practices and growth patterns among LBW infants to inform potential feeding interventions [32]. This article captures results from the qualitative sub-study, which aimed to explore the practices, beliefs, facilitators, and barriers around the feeding of LBW infants from the perspectives of mothers, family members, community leaders, HCPs, government officials, and supply chain and DHM experts in India, Malawi and Tanzania.

## Methods

### Study design

LIFE was a formative, multi-site, observational cohort study using a convergent parallel, mixed-methods design [32]. The descriptive qualitative component captured in this article used focus group discussions (FGDs) and in-depth interviews (IDIs) to understand LBW infant feeding practices, beliefs, facilitators, and barriers from the perspectives of diverse stakeholders in various settings [33–35]. Additional details on the LIFE study methods can be found in the protocol article [32].

### Study setting and population

The study was conducted in four sites in three countries in Belgaum, Karnataka State, India; Cuttack, Odisha State, India; Lilongwe, Malawi; and Dar es Salaam, Tanzania. There were six types of participants: (1) Mothers of LBW infants aged six months or less (chronological age) born with a birthweight of 1.5 kg to < 2.5 kg; (2) family members (e.g., fathers, grandmothers, aunts) of LBW infants aged six months or less born with a birthweight of 1.5 kg to < 2.5 kg who played a role in infant and young child feeding (IYCF); (3) community leaders (e.g., elders and chiefs, religious leaders, traditional healers) who advised on infant feeding or care and were viewed as influential in their communities; (4) HCPs (i.e., physicians and nurses in the study facilities where the LBW infants were born or later referred for illness, and community health workers affiliated with these study facilities but providing outpatient visits at home post-discharge) who were involved with IYCF and had been in their position for at least six months; (5) government officials who were involved with IYCF programs and/or policy-setting and had been in their post for at least six months; and (6) experts in supply chain (i.e., those providing infant feeding supplies to health facilities) and DHM (i.e., those working in HMBs or those with applied or research knowledge on what it takes to establish and run them) who had been in their position for at least six months (Table 1).

**Table 1:** Data collection methods and sample sizes

Participant type		India- Karnataka	India- Odisha	Malawi	Tanzania	Pooled
TOTAL: Focus group discussions [Number of focus groups (number of participants)]						
Mothers	Preterm LBW infants, 0–3 months	1 (5)	1 (6)	1 (4)	1 (6)	4 (21)
	Term LBW infants, 0–3 months	1 (6)	1 (6)	1 (5)	1 (5)	4 (22)
	Preterm LBW infants, 4–6 months	1 (6)	1 (6)	1 (7)	1 (6)	4 (25)
	Term LBW infants, 4–6 months	1 (5)	1 (6)	1 (4)	1 (6)	4 (21)
Family members	Male (e.g., fathers)	1 (5)	1 (6)	1 (8)	1 (5)	4 (24)
	Female (e.g., grandmothers, aunts)	1 (7)	1 (6)	2 (11)	1 (5)	5 (29)
Community leaders	Elders, chiefs, village leaders	0	0	2 (14)	1 (5)	3 (19)
	Religious leaders	0	0	1 (8)	2 (8)	3 (16)
	Traditional healers	0	0	1 (7)	1 (5)	2 (12)
	Mixed community leaders (i.e., community leaders with different roles including religious leaders and traditional healers)	2 (12)	2 (12)	0	0	4 (24)
Total		8 (46)	8 (48)	11 (68)	10 (51)	37 (213)
In-depth interviews [Number of participants]						
Healthcare providers (i.e., physicians, nurses, community health workers)		32	32	24	32	120
Total		37	35	31	39	142

†Although only one health facility in the LIFE study had a functioning human milk bank, data collectors interviewed experts in human milk banking to assess perceived barriers and facilitators to the potential use and implementation of human milk banks. LBW: Low birthweight

### Patient involvement

The design of the larger LIFE study, including this qualitative work, involved community members, clinicians, and researchers familiar with the cultural contexts of the study sites. Study tools were piloted/pretested with mothers, community members, and HCPs in all countries to ensure that interview guides were culturally appropriate, understandable, and relevant to the study population. The study team made minor edits to the study tools after pilot testing. For example, additional context was provided around the definition of ‘donor human milk’ since this term was unfamiliar in most study sites (only one facility had a HMB), and the phrase ‘low birth weight infant’ was replaced with ‘small baby’ to remove medical jargon. We used the same interview guides in all countries. All questions were created to be relevant in all settings and remain standardized across settings to enable comparison. Cultural differences between the countries were captured in the responses to the interviews.

### Data collection

Data collection occurred from July 2019 to August 2020 using semi-structured FGD and IDI guides. Trained local researchers in all sites included males and females who had qualitative research and/or clinical backgrounds and were unknown to participants. They were all fluent in English and the local language (where relevant). Efforts were made to reduce interviewer bias by providing training on qualitative data collection and the need to ask neutral questions and provide neutral responses to participant responses. Participants were recruited by local study staff through purposive sampling, either face-to-face or through email; there were no refusals to participate. All conversations took place in-person in a private location at study facilities or in the community setting (at home after facility discharge) and were audio-recorded; data collection in Tanzania occurred during the COVID-19 pandemic and local health guidelines were followed. In addition to the interviewer, a note-taker was present at all FGDs and most IDIs, and some mothers were accompanied by their infant. Participants were reimbursed for the cost of travel and lost wages.

FGDs were conducted with mothers, family members, and community leaders (Table 1). FGDs were an ideal format for these participants to explore LBW infant feeding norms, practices, and beliefs within the community; the group dynamics allowed for in-depth discussion and debate [36]. The facilitator emphasized the need for respectful disagreement as well as maintaining the confidentiality of participants. FGDs were conducted in the local language and each lasted for 90–120 min.

IDIs were completed with HCPs, government officials, and supply chain and DHM experts (Table 1). Interviews were conducted with these individuals to allow for in-depth conversations with each participant and to understand their perspectives of infant feeding based on their areas of expertise. IDIs were conducted in English or in the local language based on the comfort of the participant. IDIs were completed in 45–60 min. Data saturation [37, 38] was reached for the FGDs and other IDIs. Both the in-country interviewers and US team members agreed when data saturation was reached; new responses were no longer heard. The US and local teams had weekly check-ins throughout data collection and during analysis to discuss findings. Analysis began towards the end of data collection and after data collection was complete.

### Data analysis and management

Framework analysis was conducted [39]. We developed a codebook deductively (based on research aims and interview guides) and inductively (based on emerging themes from the interviews), which was used for all study data. Data were coded using both rapid and traditional approaches in Dedoose (Version 8.3.35). The approaches used to code the data were iterative. Team members in India and Malawi completed a rapid coding process [39–41] whereby FGD and IDI data were summarized into thematic tables soon after data collection; researchers used detailed notes and audio recordings to complete the tables. After site team members completed the thematic tables, they were reviewed by United States (US) team members and any coding and interpretation discrepancies were discussed with all interviewers and analysts. The rapid analysis approach was initially used because investigators thought it would reduce the burden on the in-country teams and generate more rapid results. During the process, the US and in-country team members regularly discussed the insights emerging from the interviews and the in-country teams felt that the learnings accurately reflected their experience with these topics and that the rapid analyses reflected what was earned during the interviews. Consequently, all team members had confidence in the rapid analysis approach. However, the time savings were minimal compared to a traditional analysis approach; and, while we were confident in the insights, we were missing out on the ability to provide quotes and additional color to go along with the insights. Based on these reflections about the process, we switched approaches for the last country (i.e., Tanzania) to capture quotes and add descriptive power. We also switched to the US team doing the analysis because of capacity constraints in the Tanzania team. The US study team members had regular conversations with local study team members about what interviewers heard during ongoing interviews and to clarify any confusion in the detailed FGD/IDI notes that were received and reviewed. FGDs and IDIs were translated and transcribed manually from Swahili to English by members of the local team. Once completed, each transcript was checked by a separate team member. Full thematic, line-by-line coding of all country data was completed by two qualitative researchers at Harvard, and interpretations/contextualization were discussed with all local researchers who conducted the FGDs and IDIs. Double coding was completed by qualitative research experts in the US (EB and LS) until consistency was reached and ensured through continued double coding of 10% of the remaining transcripts.

Data were analyzed both within and across sites to compare similarities and differences around infant feeding practices and beliefs; data were also analyzed by stakeholder type. Throughout the analysis process, the main messages for each theme were reviewed with site team members as a means to reduce analyst bias, validate the interpretations, and understand the findings within the local context. All data were stored in a password encrypted cloud server and numerical identification codes were used to protect the identities of the participants.

Numerous measures were put in place by investigators to enhance the trustworthiness of the data [42, 43]. To foster credibility of the data, we involved interviewers from the local context who had an intimate understanding of the cultural norms and beliefs as well as the local language of the participants. Interviewers spent a prolonged time with participants to establish rapport and to fully understand their perspectives. Additionally, the larger consortium included numerous study investigators who were familiar with and knowledgeable about care of LBW infants in each of the study settings. Peer debriefing was conducted among the interviewers in each country and then together as a multi-country team at the analysis stage. The US qualitative analysts discussed all results with the in-country interviewers to ensure that all interpretations were relevant to the local cultural context; this process replaced member checking, which was not deemed appropriate or routine by investigators in our study settings. Select quotations are included in the paper to share participants’ own words. While we did not apply triangulation in this paper, many of the findings resonated with our quantitative results on feeding practices in a larger moderately LBW population; these findings are published elsewhere [44]. To ensure transferability, we used the same data collection methods with various participant types in four geographical regions. In terms of dependability, the qualitative research team regularly updated the full consortium on the process of analysis and actively solicited insights and feedback from investigators who were not intimately involved with this component. Finally, confirmability was facilitated throughout the interview process whereby interviewers confirmed what they had heard and probed for any additional information.

## Results

The results capture learnings from 37 FGDs (with 213 individuals) and 142 IDIs conducted in four sites on practices, beliefs, facilitators, and barriers related to feeding LBW infants MOM and alternative feeding types. Although mother FGDs were stratified by preterm and term status, reported feeding practices did not differ between these two groups nor was a distinction made by other respondents. Minimal variation in insights was found between countries and respondents; we have highlighted any meaningful differences below. Table 2 includes frequently and less frequently mentioned themes, categorized into facilitators and barriers. Select respondent quotations, further illuminating the results shared below, can be found in Table 3. We did not include specific quotations from family members since they aligned with those of mothers and the latter were more illustrative.

**Table 2:** Facilitators and barriers to infant feeding options

		Facilitators	Barriers
Direct breast- feeding	Frequently mentioned	● Maternal nutrition (i.e., diverse diet and good nutrition) ● Lactation support ● Position of infant on breast	● Perceived insufficient milk production ● Poor latch to nipple ● Infant falling asleep ● Breast or nipple pain
	Less frequently mentioned	● Supportive home environment ● Breast milk being free/available ● Massaging breasts to stimulate milk production ● Proximity of mother to infant	● Infant vomiting or coughing ● Lack of support from healthcare providers (e.g., observations of feeding) ● Maternal stress
Expressed breast milk	Frequently mentioned	● Maternal nutrition (i.e., diverse diet and good nutrition) ● Lactation support, including physical support ● Private space	● Perceived insufficient milk production ● Breast or nipple pain ● Maternal stress ● Problems expressing by hand
	Less frequently mentioned	● Massaging breasts to stimulate milk production ● Equipment (e.g., having pumps or cups to feed) ● Observing other mothers express	● Lack of equipment in facility (e.g., cups) ● Limited staff to help caregivers ● Lack of hygiene standards for equipment
Donor human milk (DHM)	Frequently mentioned	● Community education, awareness, and outreach ● Sufficient number of donor mothers ● Trained staff	● General lack of knowledge of DHM and HMBs ● Concerns around milk safety and disease transmission ● Concerns around equipment cleanliness ● Concerns around mismatch of health, social, and religious backgrounds of donor mother and recipient infant/family
	Less frequently mentioned	● Accessibility and location of human milk banks (HMBs) for donation and distribution ● Peer support among donating mothers	● Lack of support from family and community for mothers to donate and feed breast milk ● Lack of refrigeration at the facility and home
Infant formula	Frequently mentioned	● Education on how to mix and feed infant formula ● Someone else prepares infant formula so mother does not have to	● Cost ● Knowledge/ability to prepare infant formula ● Storage ● Fear of infant illness
	Less frequently mentioned	● Infant formula provided or donated by HCP, family member, community member or well-wisher	● Access to clean water ● Confusion around which product to purchase

**Table 3:** Select quotes from LIFE study sites*

	Practices and beliefs	Facilitators	Barriers
Direct breast- feeding	“We tell [mothers] the benefits of exclusive breastfeeding because the breast milk has benefits but it reduces when mixed with other things…we insist the mothers to exclusively breastfeed the baby for the first 6 months because the mother’s breast milk is everything to the baby” -Tanzania, HCP	“Doctors told us to exclusively breastfeed for 6 months. Whenever you exclusive breastfeed but see that the baby is not gaining weight, you as a mother should try to get good nutrition, porridge, soya porridge, pumpkin seeds, eat a lot of fruits, get fresh cow milk not diluted with water so that your breast milk can be thick and satisfy the baby and grow well without feeding her anything else.” -Tanzania, Mother with preterm infant 0–3 months	“In most cases, low birth weight babies are not able to breastfeed because they become tired of feeding and their jaws are not strong enough to keep sucking the breast. They generally stop breastfeeding.” -Malawi, HCP
Expressed breast milk	“I have never seen anyone [EBM] as a matter of fact, they were shocked by me because there was a time when I had to pump milk for my baby since he was so small and couldn’t feed on his own. So they were surprised ‘we have never seen anyone doing this’ they said.” -Tanzania, Mother with preterm infant 4–7 months	“We have a special room allocated for mothers to express their milk and feed their babies…I see no problem [with privacy] since that special room only accommodates lactating mothers.” -Tanzania, HCP	“Moms may not know the technique and some have never heard of expressing and this would be their first time. They at times feel shy to do and fail to express.” -Malawi, HCP
Donor human milk	“In terms of donating the milk, that is a challenge because people have the social concept that ‘my milk is for my child’. They think about who is receiving the milk and how it is going to be helpful to them…so it is about the social concepts which we have, about expressing our milk and giving it to someone else.” -Malawi, DHM subject/practice expert	“We will first counsel them because not all mothers will be okay to feed their babies from another woman. Some will think about hygiene, diseases and other things and that is why we will first give out education on how this milk is cleaned and the state of the donors. So we will give a mother assurance as in informed consent then she will make a decision for herself. But we will counsel mothers to agree with the plan by giving them the benefits and risks of a baby lacking milk.” -Tanzania, HCP	“I won’t trust the person who conducted a test. You know he can overlook [during testing], I won’t be sure with how sterile the storage equipment was. [Those who will test the milk] are human beings, they cannot be correct in everything. I won’t trust the milk. I will have trust in my own breast milk.” -Tanzania, Mother with preterm infant 0–3 months
Infant formula	“I also learnt that formula milk causes baby diarrhea, vomiting and developmental issues.” -Tanzania, Mother with preterm infant 4–7 months	“We provide [LBW infants with formula]. Our facility is supplied with formula…we nurses prepare it.” -Tanzania, HCP	“Some women in the communities may not know how to prepare the formula because they can hardly read the instructions on the tin due to their illiteracy levels.” -Malawi, HCP

*The rapid coding and analysis process for India and Malawi limited our ability to capture verbatim quotes

EBM: Expressed breast milk. HCP: Health care provider. DHM: Donor human milk

### Mother’s own milk

Direct breastfeeding.

#### Practices and beliefs

Direct breastfeeding, defined as feeding an infant at the breast, was the predominant feeding method practiced by mothers and supported by all respondent types in all sites. Infant growth and physical and mental development were universally reported as key benefits of direct breastfeeding. Most FGD participants in all sites believed that breastfeeding provided essential nutrients for the infant. HCPs in all sites believed that breastfeeding provided immunity for LBW infants, and those in India-Karnataka thought that breastfeeding increased the quality of mother-child bonding. Only one female family member in Malawi expressed the importance of feeding colostrum to a newborn. HCPs in all sites believed that LBW infants should be exclusively breastfed for the first six months, a sentiment shared by all types of participants in all sites. However, a few mothers reported introducing other foods and liquids earlier to facilitate infant growth, reduce crying, supplement breast milk, or satiate the infant. In India, there were reports of feeding a mixture of honey and water after birth until mother’s milk came in, and in Malawi and Tanzania, some mothers fed their infant porridge or cow’s milk.

#### Facilitators

Adequate maternal nutrition, described by participants as having a diverse diet and good nutrition, and counseling by HCPs were identified as common facilitators to breastfeeding in all sites and respondent types (Table 2). Additional facilitators mentioned by mothers included repositioning the infant (India-Karnataka), a supportive home environment (India-Odisha), the convenience of breast milk being free/readily available (Malawi), and massaging breasts to stimulate breast milk production (Tanzania). HCPs and mothers in all sites reported that breastfeeding initiation varied based on when milk came in and that the proximity of the mother to the infant could make initiation happen sooner. Mothers, family members, and community leaders in India-Odisha, Malawi, and Tanzania believed that mothers should maintain a nutritious diet to facilitate breast milk production. Some mothers in the African sites shared that porridge and fruit were key components of a nutritious maternal diet; family members in Tanzania recommended meat and vegetables; and HCPs in the African sites emphasized diversity of foods and adequate liquids. Providers in Tanzania described counseling mothers on the importance of breastfeeding and stress reduction to aid in milk production. In Malawi and India, HCPs advised on breastfeeding positioning and frequency. Some HCPs further explained that they monitored LBW infants’ swallowing efforts (non-specified) and promoted skin-to-skin contact to stimulate breast milk production.

#### Barriers

In all sites, frequently mentioned breastfeeding barriers (by mothers, male and female family members and HCPs) were perceived insufficient milk production and the infant’s inability to properly attach to the nipple (Table 2). Other barriers included infants falling asleep while feeding (mothers in India-Odisha and Tanzania); breast or nipple pain, particularly engorged breasts (HCPs in all sites); infant vomiting or coughing, which required burping and feeding of smaller quantities (mothers in India-Karnataka); overcrowded wards, which made observation and counseling difficult (HCPs in Malawi); and maternal stress (HCPs in Tanzania). Mothers, family members, religious leaders, and HCPs in Tanzania cited insufficient milk production as a challenge and in some cases, felt that infant formula, animal milk, water, or porridge needed to be fed to the infant as a supplement to breast milk. HCPs believed that stress could lead to reduced production, and counseling with a focus on maternal nutrition could help address this challenge. LBW infants’ inability to suck or latch onto the nipple was another breastfeeding challenge reported by HCPs in all sites, mothers in India-Odisha and Tanzania, female family members in Tanzania, and community elders and traditional healers in Malawi. To help with this challenge, counseling from HCPs or family members was felt to be useful.

### Expressed breast milk (EBM) feeding

#### Practices and beliefs

Breast milk expression was acceptable in all sites, but practiced infrequently, especially in the community setting; direct breastfeeding was more common. Mothers most often expressed by hand into a cup (all sites); the use of a breast pump was rarely reported and only in health facilities since pumps were largely unavailable. EBM was usually fed immediately using a cup (all sites), syringe (all sites), or palladai (a cross between a spoon and cup used to feed infants in Indian sites only). Unlike in Malawi, some mothers in Tanzania and India reported storing EBM at room temperature or in a refrigerator and feeding it after a few hours. Mothers, family members, community leaders and HCPs in Malawi as well as mothers in India-Odisha felt that EBM was an appropriate option for infants who had difficulty latching or suckling at the breast. There was also a shared belief in all FGDs that EBM was useful because it could be fed by someone other than the mother, particularly when she had to attend to other duties/work. Mothers and HCPs in India-Karnataka reported that EBM was beneficial in preventing breast engorgement. An additional benefit of EBM was that caregivers could determine the amount of milk the infant consumed (HCPs in Malawi and mothers in Tanzania). Health-related and cultural beliefs formed the basis of perceived risks of EBM feeding in the community setting. Fear of illness resulting from contaminated EBM was most commonly mentioned by mothers and some family members. Some mothers and family members in the African sites worried about choking or aspiration when feeding EBM. Select religious leaders in Malawi, Ayurvedic providers (providers in India who focus on the holistic health of a person), and community leaders in India-Odisha believed EBM was less nutritious than direct breast milk. A few mothers in the Indian sites believed that an evil eye would be put on an infant who was fed EBM, and community and religious leaders felt that it would reduce mother-infant bonding.

#### Facilitators

In addition to the above-mentioned facilitators of direct breastfeeding, provision of equipment, support and space were commonly reported facilitators for EBM in all sites. Mothers in India-Odisha also found it helpful to observe others expressing breast milk (Table 2). HCPs in all sites believed that physically aiding and providing cups/syringes for EBM made the process easier. In Malawi and India-Karnataka, HCPs reported teaching mothers how to massage the breast to stimulate production and nurses in India-Karnataka physically helped mothers express and feed their infants breast milk in the neonatal intensive care unit (NICU). Having a private space to express breast milk was a frequently mentioned facilitator by HCPs in all sites.

#### Barriers

Common barriers to EBM included the perception of insufficient milk production (as reported above under direct breastfeeding), breast pain, and hygiene concerns (Table 2**)**. Similar to direct breastfeeding, perceived insufficient milk production was reported by some mothers, family members, community leaders, and HCPs in India-Odisha, Malawi, and Tanzania. Breast pain was mentioned as a barrier by mothers and HCPs in all sites, some family members in India-Karnataka and Malawi, and religious leaders in Malawi. Breast pain was described generally by mothers while HCPs in sites described pain as engorgement, retracted nipples, and sores. HCPs in all sites believed that maintaining hand and breast hygiene, cleanliness of equipment, and proper storage due to lack of refrigeration made EBM difficult. Mothers, family members, community leaders, and HCPs in all sites reported that lack of hygiene standards for manual expression and feeding vessels could lead to infant illness (e.g., diarrhea). HCPs proposed solutions for maintaining cleanliness such as teaching caregivers how to clean feeding utensils (Malawi) and discarding unused milk (Malawi and India-Karnataka). Mothers in India-Odisha and Tanzania reported difficulties with physical hand expression of milk; and HCPs shared facility-level barriers, including lack of cups (Tanzania), limited staff to assist mother-infant dyads in the NICU (India-Karnataka and Tanzania) and high cost in procuring breast pumps (Malawi).

### Alternative feeding types

Donor human milk (DHM).

#### Practices and beliefs

DHM was a new concept for most participants so responses are based on theoretical rather than actual use; India-Karnataka was the only site with an HMB, therefore, some participants from Karnataka were more familiar with the concept. In general, mothers in all sites indicated that they would be more willing to donate their breast milk rather than to feed DHM to their own infants, but shared concerns around maintaining milk supply for their own infant when donating. While most mothers indicated no expectation of an incentive for donating, describing it as a “noble act” (India), some felt that provision of extra food would be helpful to assist with milk production (Malawi). HMB experts in the African sites believed that caregivers would accept DHM to facilitate infant growth and survival when breast milk was not available or delayed. Similarly, religious and community leaders in Malawi felt that DHM could be a good option if it were more affordable than infant formula. HCPs and some family members in both Indian sites, as well as DHM experts in the African sites, stated that DHM was a better option than infant formula because it was human milk. In terms of establishing an HMB, participants in all sites felt strongly that community- and facility-based individuals as well as government officials should be involved to assist with education, logistics, and policies. DHM experts and male family members felt that more mothers would be willing to donate milk if they knew that their milk would benefit an infant in need.

#### Facilitators

Respondents suggested that community awareness and health system inputs, such as trained staff and HMB maintenance, could facilitate human HMB establishment, acceptance and uptake (Table 2**).** Since DHM was a new concept in most sites, the majority of participants felt that robust outreach and education on its benefits, accessibility, and use to all stakeholders (e.g., donating and receiving mothers, HCPs, government officials, and community members) was needed to achieve acceptance. To facilitate the successful establishment and implementation of an HMB, participants highlighted the need for demand generation and maintenance (e.g., through community engagement and peer counseling by previous donors) and logistics (e.g., cold chain and training of staff).

#### Barriers

A general lack of knowledge, concerns about milk safety, the donor’s profile, and family resistance were reported as common barriers to DHM acceptance and potential future uptake (Table 2**)**. While rare, all types of participants shared various misconceptions, including a lack of regulation of HMBs, availability of DHM based on affordability rather than need, and DHM serving as a barrier to breastfeeding. In all sites, participants expressed a general concern around safety related to milk collection, pasteurization, and storage. Many mothers and HCPs in all sites worried about potential transmission of HIV, Hepatitis B, diarrhea and other infections. In all sites, the donor’s health and social background were reported as potential barriers. In India, HCPs and religious leaders were concerned about the mismatch of caste and religion between the donor and recipient of the milk. Mothers in African sites worried that certain traits and/or illnesses of the donor could be passed through the milk to the receiving infant. Further, mothers in all sites and HCPs in India mentioned family (unspecified) resistance towards feeding DHM as a barrier, and some participants in Malawi warned that in spite of community awareness, resistance could persist in rural communities due to skepticism regarding research and policies related to infant feeding.

### Infant formula

#### Practices and beliefs

In general, all participant types in all sites were against the use of infant formula unless absolutely necessary (i.e., mother-infant separation or maternal death). In rare instances when infant formula was provided, it was usually fed as a supplement to breast milk to encourage growth and save an infant’s life, rather than a replacement feed.

#### Facilitators

Among mothers who fed infant formula to their LBW infants, only a few facilitators were mentioned. For example, HCPs across all study sites felt that teaching mothers how to mix and feed infant formula was helpful. Similarly, having a family member or HCP prepare the infant formula facilitated its use. Although rare, the provision or donation of infant formula by a HCP, family member, community leader, or a well-wisher/organization facilitated access to infant formula for mothers and caregivers who needed it.

#### Barriers

Lack of affordability, knowledge, maintenance of hygiene during preparation, storage of infant formula, and fear of illness were commonly reported barriers to infant formula feeding (Table 2). Additionally, some family members in Tanzania reported confusion about which infant formula products to buy and beliefs that fake products were being sold. Many mothers, family members, and HCPs in all sites reported that the cost of infant formula was burdensome. In rare cases, some mothers in India-Odisha heard of others diluting infant formula to make it last longer. HCPs in Tanzania felt that understanding the right proportions of water to powder could be difficult for mothers, especially with changing infant needs over time. HCPs reported that some mothers in Malawi had limited literacy and were unable to read the instructions on infant formula containers. Finally, respondents from all participant types in all sites perceived that infant formula feeding could lead to infant illness, including diarrhea, constipation, digestive issues, vomiting, and necrotizing enterocolitis (HCPs only), if infant formula was not prepared correctly or hygienically or if the infant’s intestines were not mature enough to digest it.

## Discussion

This qualitative study explored the practices, beliefs, facilitators, and barriers related to LBW infant feeding in the first half of infancy in South Asia and sub-Saharan Africa among multiple stakeholders. All participants believed that MOM was best for LBW infants and most felt that it should be fed exclusively up to six months. This aligns with WHO global recommendations for optimal infant feeding and is consistent with existing evidence among NBW infants in LMICs [30, 45–50]. Direct breastfeeding was most commonly practiced; EBM was also deemed acceptable but less common. Infant formula feeding was rare, and DHM was a new concept for most participants. Some mothers reported feeding commercial milk formula or other liquids because they felt their breast milk was insufficient, a practice commonly cited in other studies [51, 52]. Overall, perspectives of various stakeholders aligned, which could be explained by family and community dynamics as well as hierarchies within the health system.

Our study uncovered barriers relevant to the immaturity of LBW infants, such as poor latching ability and tiredness when breastfeeding. Many of the other findings were similar to those revealed in previous studies conducted predominantly among NBW infants [46, 53, 54]. Of note, our sample included moderately LBW infants who may not be perceived to be as vulnerable as very LBW infants often requiring advanced medical support. Studies in India and Uganda showed that infant size was not easily recognized by mothers as a risk factor for poor health, especially without direct comparison to other children [25, 55]. Community perceptions of infant size could influence feeding decisions and care-seeking behaviors. Community awareness around the vulnerability of LBW infants and the need for specialized care could enhance outcomes long term.

Participants in our study believed adequate maternal nutrition and lactation support from HCPs facilitated feeding MOM, similar to other studies conducted among NBW infants that assessed perceptions of maternal diet and milk production [30, 45–49]. However, some mothers faced challenges with breast milk feeding, including perceived insufficient milk production, infant’s inability to attach to the nipple, and mother-infant separation after birth in the facility; our findings were consistent with other studies [29, 46, 49, 52, 56]. In the community setting, mothers in our study felt that their LBW infants were not satisfied with their breast milk alone, which contributed to the introduction of food or liquids prior to six months of age; the WHO recommends that an infant be exclusively breastfed up to six months of age. This finding has also been noted in other studies by mothers of NBW infants [25, 46, 49, 56]. Unlike mothers, HCPs reported that milk production was adequate, highlighting the need for lactation education and support for mothers and caregivers of LBW infants [49]. Benefits of EBM, such as increased milk supply and ability to continue feeding MOM despite latch challenges and separation from the infant, should be emphasized for mothers and families of LBW infants. Additionally EBM could alleviate breast pain and engorgement, noted as common barriers to direct breastfeeding [30, 45, 46, 56, 57].

While HCPs in our study reported providing counseling on breastfeeding positioning and emotional support, more consistent and targeted support for LBW infants is needed in the facility and community. Currently available resources or counseling guidelines, including those on EBM and DHM, are geared towards infants in high-income settings or tailored towards healthy and/or term infants [58–60]. Additional areas of focus in specialized lactation support should include early breastfeeding initiation and the nutritional value of colostrum for LBW infants as its importance was rarely recognized by participants in our study. Overall, our findings suggest that water, sanitation and hygiene (WASH) practices were barriers to alternative feeding options. In particular, the availability of safe, clean water for infant formula preparation and maintenance of hand and breast hygiene during hand expression were felt to be challenges; this suggests the need for comprehensive feeding counseling that addresses safety and cleanliness of water, hands and feeding tools. Improving WASH is particularly relevant for preterm and LBW infants who may need assistance with feeding through the use of cups, nasogastric tubes, or other vessels. Additional research is needed to determine how to effectively tailor WASH interventions for LBW infants at the facility and household level.

Although DHM was a new concept for most participants and HMBs were not yet present in most sites, our findings signal that it could be a viable feeding option for LBW infants who do not have access to MOM. However, the concerns that were raised, such as hesitation to feed infants with DHM and fear of disease transmission, must be carefully considered and addressed as noted in similar studies [47, 51, 61]. In order to allay concerns, participants in our study reported that community education and awareness would be critical to foster future acceptance and uptake of DHM. Researchers in Uganda found that DHM acceptability was contingent on buy-in from the entire community and key facility staff rather than through a top-down approach via policy mandates [51]. Further, the feasibility of DHM use might vary by site based on availability and political buy-in for HMBs; for example, milk banking is spreading rapidly in India while uptake is slower in the African sites [62, 63]. Understanding sociocultural factors will play a vital role in the future recruitment of milk donors and awareness-building for caregivers of LBW infants in need of DHM [64]. Lastly, DHM is not meant to be a replacement for MOM but rather could serve as a bridge to feeding MOM before it becomes available or sufficient. Before mother-infant dyads are discharged from the facility, they should receive tailored lactation support so they can effectively breastfeed their LBW infant [65].

This study had several strengths. It shared perspectives of stakeholders at the household, community and health system level and those involved in providing and supporting LBW infant feeding. In most communities, feeding decisions were not solely left to the mother; rather, she was influenced by her family, community, and health system. Moreover, this study was conducted in four sites in two geographically diverse regions, representing the highest rates of LBW births and child deaths. Learnings from this work can provide guidance on the facilitators, barriers, and key stakeholders to target in behavior change in the community and quality improvement at the facility-level to promote optimal LBW infant feeding.

There were some inherent limitations. The rapid coding approach meant having direct quotes only for a subset of our results (as evidenced in Table 3), but this technique enabled us to have frequent conversations with the site-based qualitative researchers to interpret data closer to real-time [37, 66]. As a group, we were able to determine if there were topics that needed to be explored more deeply in subsequent FGDs and IDIs. We also acknowledge that LBW infants in this study lived in predominantly urban settings and had improved access to care, potentially limiting the generalizability of the results to a more rural population. It was also challenging to capture the nuance within our findings for the facility versus community setting. Lastly, we collected data at a single time point for each mother-infant dyad and were unable to evaluate changes in practices, beliefs or experiences throughout the first half of infancy.

## Conclusion

Given that the existing evidence on experiences of infant feeding has been focused primarily on NBW infants, this study adds important considerations for the feeding of LBW infants in South Asia and Sub-Saharan Africa. It is encouraging that direct breastfeeding is predominant among all infants alike. Yet, despite reported concerns around feeding difficulties and perceived insufficient milk, EBM was rarely fed; a missed opportunity for LBW infants and their mothers. Efforts are needed to promote and support EBM as a mechanism to address feeding difficulties, increase milk production, and enhance milk volume consumed. Design and provision of specialized lactation support and feeding counseling for LBW infants can address numerous obstacles faced by mothers, infants and the health system. Such a comprehensive package can serve to enhance breast milk feeding initiation, duration and quality, thereby improving overall health outcomes in both the mother and infant. Provision of verbal, physical and emotional support to mothers and family members can increase knowledge and empower the performance of optimal practices, debunk misconceptions, decrease stress, and prevent or resolve feeding difficulties. Observation of and support to LBW infants can help identify and overcome common and unique feeding challenges before they are exacerbated. Finally, buy-in from facility leadership and training of facility staff can serve to create the policy and infrastructure changes needed to promote family-centered care, including lack of separation between mothers and infants, provision of universal and consistent lactation support and feeding counseling throughout the hospital stay, and space for breast milk expression and feeding. Finally, although DHM was a new concept for most, the overall positive attitudes and insights for this specific vulnerable population will be instrumental in the development and scale-up of HMBs to serve as a bridge to the provision of MOM.

### Electronic supplementary material

Below is the link to the electronic supplementary material.

Supplementary Material 1

## Data Availability

The datasets generated and/or analyzed during the current study are not publicly available due to the nature of this qualitative research and the potential to identify respondents but are available from the corresponding author on reasonable request.

## Abbreviations

DHM: Donor human milk
EBF: Exclusive breastfeeding
EBM: Expressed breast milk
FGD: Focus group discussion
HCP: Health care provider
HMB: Human milk bank
IDI: In-depth interview
IYCF: Infant and young child feeding
LBW: Low birthweight
LIFE: Low Birthweight Infant Feeding Exploration Study
MOM: Mother’s own milk
NBW: Normal birthweight
NICU: Neonatal intensive care unit
WASH: Water, sanitation and hygiene
WHO: World Health Organization
