# Supplementary material for: Facilitators, barriers, and key influencers of breastfeeding among low birthweight infants: a qualitative study in India, Malawi, and Tanzania
Source: Int Breastfeed J. 2023 Nov 8;18:59. doi: 10.1186/s13006-023-00597-7 (PMC10634072; doi:10.1186/s13006-023-00597-7)
Supplement: Supplementary file 1 — Supplementary Material 1 [file 13006_2023_597_MOESM1_ESM.pdf]

SUPPLEMENTARY MATERIAL

Facilitators, barriers and key influencers of breastfeeding among low birthweight infants: a qualitative study in India, Malawi, and Tanzania

Supplement 1. Focus group discussion guide for mothers
Supplement 2. Focus group discussion guide for family members
Supplement 3. Focus group discussion guide for community leaders
Supplement 4. In-depth interview guide for healthcare providers
Supplement 5. In-depth interview guide for government officials
Supplement 6. In-depth interview guide for donor human milk bank experts
Supplement 7. In-depth interview guide for supply chain experts

Supplement 1. Focus group discussion guide for mothers

MOTHERS FGD

**SECTION I: Beliefs and attitudes around IYCF**

Question 1: There are different ideas on what causes a baby to be small. What you think causes a baby to be born small? Why do you think that?

a. Does your family or community have other ideas about what causes babies to be born small? What do they think?

Probe for both FGD groups

1. *Are there any community beliefs? Religious?*

Question 2: I'd like to hear about what you feed your baby. We know that there are lots of different foods and liquids that babies are fed, and different ways of giving these foods and liquids to a baby. Please describe all of the different things or ways that you feed your small baby.

For FGD with moms with babies 0-3 months ask:

a. What did you feed your baby the day he or she was born?
b. During your baby's first month, have you changed what you feed him or her? How has it changed? Why did you make this change?

For FGD with moms with babies 4-6 months ask: After your baby was a few months old, what did you feed him or her?

Probes for both FGD groups

1. *Do you give your babies food or liquid other than breast milk? At what age? What do you feed them? Why did you decide to feed your baby that?*
2. *Does someone else ever feed your baby? When? Who (role) is this person? What do they feed your baby?*

Question 3: If a small baby is having trouble growing, what do you think could help the baby grow? Why do you think that?

Probes for both FGD groups

1. *How often do you feed your baby if s/he isn't growing well?*
2. *Do moms in your community change their diet at all if a baby is having trouble growing? How (what changes do they make)?*

Question 4: During the first six months, what do moms in your community feed their small baby if they're sick, for example with fever or diarrhea?

Question 5: Where do you get information or advice on what to feed your small baby?

a. When different people give you advice, whose advice do you follow?

Probes for both FGD groups

1. *Do you get advice from: Clinicians? Family members? Friends? Community/elders? Traditional healers? Internet/Apps/TV/radio?*

**SECTION II: Facilitators and barriers to IYCF**

Question 6: What, if anything, makes it hard to feed your small baby?

a. How do you handle that challenge(s) that you mentioned?

b. When you are outside of your home going about your daily activities, are there places where you can breastfeed or express breast milk? Expressing breast milk means when a woman releases milk from her breast either by hand or with a pump.
    i. Is doing this difficult or easy? Why?

Question 7: It sounds like there are a few challenges that you mentioned. Can you please share what you have found helps to feed your small baby?

**SECTION III: Feasibility and acceptability of IYCF options**

For this next set of questions, we're going to discuss different ways to feed small babies in addition to giving breast milk. I am going to ask what you think about formula and feeding babies milk from another woman. We're asking these questions because we want to hear about your opinions and experiences with these ways of feeding babies so we can better understand if these options could help small babies grow faster. Does anyone have any questions?

Question 8: How do you feel about feeding a small baby formula?

a. Do you think there could be benefits to feeding a small baby formula? What are they?
b. Do you think there could be risks to feeding a small baby formula? What are they?
c. What do you think could be some challenges when feeding a baby formula?
d. What do you think could make it easy to use formula?

Probes for both FGD groups

1. *Would access to clean water for mixing formula be a challenge?*
2. *Would the cost of formula be a problem?*
3. *Do you think there would be any family pressure to feed or not feed a low birthweight baby formula?*
4. *Has anyone learned how to prepare formula? If so, how did you learn to prepare it?*

Question 9: Do moms in your community express breast milk?

a. If yes: Why do they express breast milk?
b. If no: Why do moms not express breast milk?
c. What do you think would be bad or difficult about feeding a small baby expressed breast milk?
d. What do you think could be some good things about feeding a small baby expressed breast milk?

There are times when a mom is not able to breastfeed her baby, make enough milk, or is not available to breastfeed her baby. In this case, sometimes babies will have milk that is donated from a woman that the mom does not know. This is different from moms sharing their breast milk with each other or breastfeeding each other's baby. To make sure the milk is safe for drinking, the milk is tested and cleaned. The donated milk might be fed to the baby in a special cup, spoon, or palladai. Donor human milk and milk facilities/banks already exist in some countries. Does anyone have any questions about this?

Question 10: Based on what we just described, what do you think about feeding small babies donated breast milk? Why do you think that?

a. What do you think could be some good things about feeding a small baby donor human milk?
b. What do you think could be some bad things about feeding a small baby donor human milk?

Question 11: If there was a human milk facility in your community and you had a way to express breast milk, do you think you would donate breast milk to feed other babies? Why or why not?

a. How do you think your family members would react to you donating breast milk? Why?

b. Would you expect a gift to donate breast milk?

Probes for both FGD groups

1. *Would you donate if you were making more milk than you needed for your baby? Why or why not?*

Question 12: If it might help your small baby grow faster, would you allow your baby to be fed donated breast milk? Why or why not?

a. How do think your family and community would feel about you feeding your baby donated breast milk?

SECTION IV: Feasibility and acceptability of an IYCF intervention

The last topic that I want to discuss with you is the possibility of moms and small babies volunteering to be part of a study to help babies grow better and faster.

Question 13: If you were in the study, how would you feel about coming into the clinic with your baby when your baby is healthy and doing well? Why?

Question 14: The study may also include taking small amounts of blood or breast milk from the mom and the baby. How do you think people in your community would feel about giving small amounts of blood and breast milk for a study? Why do you think they would feel this way?

a. Would moms expect a gift for doing this? What could it be?

CLOSING

Question 15: Before we finish, what advice do you have for moms with small babies?

Supplement 2. Focus group discussion guide for family members

FAMILY MEMBER FGD

**SECTION I: Beliefs and norms around IYCF**

Question 1: In general, how do moms in your family feed their small baby:

a. During the first week?
b. During the first month?
c. During the first six months?

Question 2: If a small baby is having trouble growing, what do you think could be done to help the baby grow? Why?

Probes

1. *How often does your [wife/sister/daughter/daughter-in-law/niece/granddaughter] feed your baby if she or he is having trouble growing?*
2. *Do moms in your family change their diet at all? How?*

Question 3: During the first six months, what do people in your family feed babies if they're sick?

Question 4: Are you directly involved with feeding your small baby during the first six months?

a. If yes: Tell me about how you are involved. How do you feed your baby? What do you feed the baby? How often do you do this?
b. If no: Why aren't you involved with feeding your baby?
c. For all: If at all, what makes it hard for you to be involved in feeding your baby?
    i. How do you handle that challenge?

Question 5: Are there any other beliefs in your community about how to feed a baby from 0-6 months? Why do people think that?

**SECTION II: Feasibility and acceptability of IYCF options**

For this next set of questions, we're going to discuss different ways to feed small babies in addition to giving breast milk. I am going to ask what you think about formula and feeding babies milk from another woman. We're asking these questions because we want to hear about your opinions and experiences with these ways of feeding babies so we can better understand if these options could help small babies grow faster. Does anyone have any questions?

Question 6: How do you feel about a small baby being fed formula in the first six months of life?

a. Do you think there could be benefits to feeding a baby formula? What are they?
b. Do you think there could be risks to feeding a baby formula? What are they?
c. What do you think could be some challenges when feeding a baby formula?
d. What do you think could make it easy to use formula?

Probes for potential challenges:

1. *Would access to clean water for mixing formula be a challenge?*
2. *Would the cost of formula be a problem?*

Probes for potential facilitators:

1. *If anyone says that they use formula: how did you learn how to prepare it?*

Sometimes moms will express their breast milk to feed their baby instead of breastfeeding. By expressing breast milk we mean when a woman releases milk from her breast either by hand or with a pump.

Question 7: Do moms in your family express breast milk?

a. If yes: Why do moms express breast milk?
b. If no: Why do moms not express breast milk?
c. What do you think could be bad or difficult about feeding a small baby expressed breast milk?
d. What do you think could be some good things about feeding a small baby expressed breast milk?

There are times when a mom is not able to breastfeed her baby, make enough milk, or is not available to breastfeed her baby. In this case, sometimes babies will have milk that is donated from a woman that the mom does not know. This is different from moms sharing their breast milk with each other or breastfeeding each other's baby. To make sure the milk is safe for drinking, the milk is tested and cleaned. The donated milk might be fed to the baby in a special cup, spoon, or palladai. Donor human milk and milk facilities/banks exist in some countries. Does anyone have any questions about this?

Question 8: Based on what I just described, what do you think about feeding small babies donated breast milk? Why do you think that?

a. What do you think could be some good things about feeding a baby donor human milk?
b. What do you think could be some bad things about feeding a baby donor human milk?

Question 9: If [your baby/a baby in your family] could not have their mom's own milk, would you allow [your baby/a baby in your family] to drink donated human milk? Why or why not?

Question 10: Would you allow your [wife/sister/daughter/daughter-in-law/niece/granddaughter] to donate her milk for infants that can't have their own mother's milk? Why or why not?

Probes

1. *Are there community or cultural beliefs that exist around sharing milk?*
2. *What would you need to know about donating human milk before having your [wife/sister/daughter/daughter-in-law/niece/granddaughter] donate her milk?*

SECTION III: Feasibility and acceptability of IYCF intervention

The last topic that I want to discuss with you is the possibility of moms and babies like the ones in your family volunteering to be part of a study to help babies grow faster.

Question 11: If your family member were in the study, how would you feel about coming into the clinic when the baby seems to be healthy and growing well? Why?

Question 12: The study may also include taking small amounts of blood or breast milk from the mom and baby. How would you feel about your [wife/sister/daughter/daughter-in-law/niece/granddaughter] and baby giving small amounts of blood or breast milk for a study? Why do you feel this way?

CLOSING

Question 13: Before we finish, what advice do you have for [dads/family members] with small babies?

Supplement 3. Focus group discussion guide for community leaders

COMMUNITY LEADERS FGD

**SECTION I: Beliefs and attitudes around IYCF**

I'd like to start the conversation by hearing about the different ways that women feed their low birthweight babies. In particular, we'd like to understand the role that community leaders play in how mothers feed their babies.

Question 1: Please describe the situations in which a mother or someone in her family would typically come to you for advice on feeding her baby.

a. What kind of advice do you offer moms or their families about feeding their babies for 0-6 months?

Probes

1. *What might be happening with the baby or the mother in terms of their health?*
2. *Do mothers come for advice when the baby is healthy and feeding well?*

Question 2: Have you ever had any experience with providing guidance on feeding small babies?

a. If yes: What you think is the best way to feed a small baby for the first six months of life? Why do you think is this best for the baby?
b. If no: skip to Q3

Question 3: If a small baby is having trouble growing, what do you think could be done to help the baby grow? Why?

For religious leaders only: When a mom has a new baby, are there any religious traditions related to what she feeds her small baby in the first six months? What are they?

Potential probes

Probes if not answered through question

1. *How often should a mother feed her baby if she or he isn't growing well?*
2. *Are there certain foods you would suggest feeding the baby? Are there certain medicines?*
3. *Would you suggest that a mother change her diet at all? How?*

Question 4 for Community and Religious Leaders: If a mom or someone in her family came to you because they had received different advice from her doctor and traditional healer about how to feed her small baby, what would you do? Why?

Question 4 for Traditional Healers: Do you know what doctors and nurses at the health facilities recommend for how to feed low birthweight babies 0-6 months?

a. If yes: How, if at all, are your recommendations for feeding a small baby different from what they recommend for months 0-6? Why are they different?
b. If yes: If your advice is different from a doctor's or nurse's, do you know whose advice does mom typically follows? Why do you think she does that?

If no: skip to Q5

Question 5 for Traditional Healers: Can you tell me about any beliefs in traditional medicine that exist around:

a. Breastfeeding?
b. Colostrum or the first breast milk?
c. Shared milk?

d. Babies who have trouble breastfeeding?
e. Solid foods in the first six months of life?

SECTION II: Feasibility and acceptability of IYCF options

For this next set of questions, we're going to discuss different ways to feed small babies in addition to giving breast milk. I am going to ask what you think about formula and feeding babies milk from another woman. We're asking these questions because we want to hear about your opinions and experiences with these ways of feeding babies so we can better understand if these options could help small babies grow faster. Does anyone have any questions?

Question 5: How do you feel about a baby being fed formula for the first six months of life? Why do you think that?

a. Do you think there could be benefits to feeding a baby formula? What are they?
b. Do you think there could be risks to feeding a baby formula? What are they?

Sometimes moms will express their breast milk to feed their baby instead of breastfeeding. By expressing breast milk we mean when a woman releases milk from her breast either by hand or with a pump.

Question 6: Have you ever heard of women doing this? What are your opinions on moms doing this to feed their babies in the first six months of life?

There are times when a mom is not able to breastfeed her baby, make enough milk, or is not available to breastfeed her baby. In this case, sometimes babies will have milk that is donated from a woman that the mom does not know. This is different from moms sharing their breast milk with each other or breastfeeding each other's baby. To make sure the milk is safe for drinking, the milk is tested and cleaned. The donated milk might be fed to the baby in a special cup, spoon, or palladai. Donor human milk and milk facilities/banks exist in some countries. Does anyone have any questions about this?

Question 7: If there was a human milk facility in your community, how would you feel about it? Why do you think that?

a. How would you feel about a small baby being fed donated human breast milk? Why?

Question 8: When, if at all, would you advise a mom to feed her baby with milk from a donor human milk facility? Why?

Question 9: What do you think some of the benefits might be of having a milk facility in your community?

Question 10: Tell me about some challenges that you think might occur if you had a milk facility in your community.

a. For challenge: how do you think we might address or avoid this challenge?

Potential probes

Probes if not answered through question

1. *Are there cultural challenges? Religious challenges? Logistical or financial challenges?*

Question 11: Thinking of your community, who do you think we would need approval from to set up a human milk facility? Is there anyone that we should definitely talk to?

Probes: religious leaders, opinion/community leaders, elders (non-governmental individuals)

SECTION III: Feasibility and acceptability of an IYCF intervention

We're working with people who are trying to figure out the best way to feed LBW babies to help them grow faster. As part of this, doctors, nurses and researchers will be trying to understand how the babies grow when they are fed in different ways. The study may recommend feeding options that are different than what you recommend.

Question 12: What role do you think [community leaders/religious leaders/traditional healers] could play in supporting or developing the study?

a. What would you want to know about the feeding options in the study to decide about whether or not you could support them?
b. What role do you think they could play to support or encourage the use of the different infant feeding options?

CLOSING

Question 13: Is there anything we didn't talk about today that you think we should know?

Supplement 4. In-depth interview guide for healthcare providers

HEALTHCARE PROVIDER IDI

**SECTION I: Current standard of care for low birthweight infants**

Question 1: First, please tell me what is your role at your facility.

Now I'd like to start discussing your experience with caring for low birthweight babies and the current standard of care for these babies.

Question 2: How care plans are formed for low birthweight babies at your facility? To start, please describe a typical care plan for these babies.

a. How is it determined whether or not a low birthweight newborn is brought to the NICU?
b. Do you do kangaroo care/skin-to-skin at your facility?
   i. If yes: Tell me about how and when this is done.
   ii. If yes: How is it determined whether or not a low birthweight newborn is uses kangaroo care?
c. If at all, how does the care plan differ for low birthweight preterm and low birthweight full-term babies? Preterm here is defined as any baby born before 37 weeks.

Question 3: Thinking of the care plan that you just described, under what circumstances would that plan look different?

Potential probes

Probes if not answered through question

1. *Anything related to mom or baby's health? (baby needed ventilation, had surgery or illness)?*
2. *Staff availability, resource constraints?*
3. *Differences in care when baby is in NICU or regular floor?*

Question 4: Once a mom and her low birthweight baby are discharged, what, if any, postnatal care is recommended?

a. How often would you say the recommended plan is followed? What are some reasons why the plan may not be followed?
b. In general, where do these visits occur (in facility or at home)?
c. What do you discuss?

Question 5: Overall, how satisfied are you with the care that low birthweight babies receive at your facility? Why?

**SECTION II: Infant feeding recommendations**

Thank you for sharing all that you have so far. Next, I'd like to hear about different infant feeding recommendations you might give to moms.

Question 6: In general, what advice do you give to moms for feeding their low birthweight baby during the baby's first few weeks?

Probes if not answered through question

1. *What advice do you give if the baby is sick with a fever? Sick with diarrhea? Other common illnesses that occur among low birthweight babies?*

2. *What advice do you give if the baby is not feeding well and losing or not gaining weight? Are there vitamins or minerals you recommend? What are they?*
3. *What advice do you give if the baby is latching and feeding well but still not gaining weight? Are there vitamins or minerals you recommend? What are they?*

Question 7: If a mom isn't producing enough milk or isn't able to breastfeed, what do you generally recommend?

a. What kind of lactation support is available to these moms to increase their milk supply?
    i. Is there any emotional support available for these moms?

SECTION III: Feasibility and acceptability of IYCF options

For this next set of questions, we're going to focus on different ways to supplement breastfeeding low birthweight babies. Specifically, I'm going to ask about your views on formula and donor human milk. We'll be using this information to help inform a revised approach for feeding these babies. We already have some ideas on what may be different ways to feed these babies but we would like your input to help us better understand why these options may or may not work. Some of the questions are very specific and that's because we're looking for your help as we figure out the details of the revised feeding strategy and what would be important for using these strategies in your facility. Do you have any questions?

Question 8: If a low birthweight baby is exclusively breastfed but is losing or not gaining weight in the first few weeks of life**,** how do you feel about the baby being fed formula ? Why do you think that?

a. How do your recommendations differ for full-term vs. preterm low birthweight babies?
b. What do you think are the benefits to feeding low birthweight babies formula?
c. What do you think are the risks to feeding low birthweight babies formula?
d. What do you think are the challenges to feeding low birthweight babies formula?
e. Are there vitamins or minerals you recommend if a baby is being fed formula? What are they?

Probes for challenges

1. *Probe for cost of formula, storing formula, and preparing formula*

Sometimes moms will express their breast milk to feed their baby instead of breastfeeding. By expressing breast milk we mean when a woman releases milk from her breast either by hand or with a pump.

Question 9: When moms of low birthweight babies are at your facility, do they express breast milk? Tell me more about how they express breast milk.

a. Ask if above is yes or no: Is any advice given to moms about how to express breast milk? If yes: who gives advice? What is the advice?
b. What do they do with the expressed breast milk? Do they feed it to their baby or throw it away?
c. Is there anything about being in the facility that could make it difficult for moms to express breast milk? (i.e. challenges if baby is in the NICU or with mom; privacy)

I'd now like to discuss a different way that moms may feed their low birthweight baby. Have you heard of donor human milk and milk banks?

If participant has not heard of donor human milk then say: I'd like to share some information with you. There are times when a mom is not able to breastfeed her baby, make enough milk, or is not available to breastfeed her baby. In this case, sometimes babies will have milk that is donated from a woman that the mom does not know. This is different from moms sharing their breast milk with each

other or breastfeeding each other's baby. With donor human milk, women donate their milk. To make sure the milk is safe for drinking, the milk is tested and cleaned. The donated milk might be fed to the baby in a special cup, spoon, or palladai. Donor human milk and milk facilities/banks exist (**INDIA ONLY say**: here and in other countries) in other countries. Do you have any questions about this?

Question 10: As part of a strategy for feeding low birthweight babies, how would you feel about having a donor human milk facility at your hospital? Why do you think that?

a. What kind of information would you want to know?

Probes if not answered through question

1. *Whose support would be needed?*
2. *What kinds of challenges do you think there could be?*
3. *Can you think of any policies would need to be put in place?*
4. *Do you think a donor human milk bank could be sustained over time? Why?*

Question 11: If there was a donor human milk bank in your facility, how would you feel about recommending donor human milk for low birthweight babies in the baby's first few weeks of life? Why?

a. What do you think are the benefits of using donor human milk?
b. What do you think are the risks of using donor human milk?
c. What kind of challenges do you think there might be when using donor human milk?
d. Are there vitamins or minerals you recommend if a baby is being fed donor human milk? What are they?

Question 12: Is there anything else that you think we should know about feeding low birthweight babies formula or donor human milk?

SECTION IV: Feasibility and acceptability of an IYCF intervention

Question 13: During today's conversation, we talked about breastfeeding and expressing breast milk, formula feeding, and donor human milk. We realize that there may be other ways that babies can be fed successfully. As our team thinks about how to best help low birthweight babies, is there anything else that you would recommend for feeding the feeding strategy? What advice do you think would be useful for us to know?

Supplement 5. In-depth interview guide for government officials

GOVERNMENT OFFICIAL IDI

**SECTION I: Commitment to LBW infant feeding strategies**

Question 1: Please tell me about your role at the [say relevant government entity].

Question 2: Do you have any policies that focus on low birthweight babies? By low birthweight, I mean babies that are born less than 2.5 kilograms or small for their age.

a. If yes: Can you please provide a couple of examples of these policies?
b. If yes: Are they part of a larger health strategy? Tell me more about that.
c. If either yes or no: In your opinion, how much of a priority are policies or programs related to low birthweight babies? Why?
d. If no: Do you have any policies that focus on the health of newborn babies? What are they?

Probes

1. *Probe for policies related to breastfeeding, formula, and vitamins for low birthweight babies*

Question 3: In your opinion, are these guidelines adequately implemented in all health facilities? Why or why not?

a. How are health facilities held accountable for implementing the policies that you mentioned?

Probes

1. *Who needs to be involved with upholding policies?*

**SECTION II: Possible risks and their mitigation**

We would like to test different infant feeding strategies to help low birthweight infants who are not growing and gaining weight similar to other infants of their age. In addition to breastfeeding, there could be recommendations around giving formula, milk from other women, or vitamins or minerals. Moms and babies would be enrolled in the study and followed for a certain period of time. As part of the study, we might need to work with health facilities and local governments.

Question 4: How would you see your role fitting into a study like this?

a. What would you need to know in order to support a study like this?
b. What could be some factors that would make you concerned about supporting a study like this?

Question 5: Who are the individuals that need to be included:

a. Before the study starts?
b. During study implementation?

Question 6: What do you think could make a study like this successful?

Question 7: What challenges could you see occurring at the local level for a study that tests different ways to feed low birthweight babies?

a. How do you think we could address these challenges?

Question 8: The different feeding options that we might test could include using baby formula and treated water. Thinking of the supply chain in your country, how do you think we can ensure a stable supply of those resources?

a. Can you describe any challenges that occur in the supply chain?

Question 9: Thinking of other competing projects, how much of a priority would a study that could potentially help low birthweight babies grow be for your country? Why?

a. What would be needed to make this a top priority?

SECTION III: Views on donor human milk, milk banks, and formula

Question 10: How do you think low birthweight babies who cannot have their mother's own milk should be fed? Why?

a. How would you feel about a mom feeding her low birthweight baby formula? Why?
b. How would you feel about a mom feeding her low birthweight baby milk from another woman? Why?

I'd now like to discuss a different way that moms may feed their low birthweight baby. Have you heard of donor human milk and milk banks?

If participant has not heard of donor human milk then say: I'd like to share some information with you. There are times when a mom is not able to breastfeed her baby, make enough milk, or is not available to breastfeed her baby. In this case, sometimes babies will have milk that is donated from a woman that the mom does not know. This is different from moms sharing their breast milk with each other or breastfeeding each other's baby. With donor human milk, women donate their milk. To make sure the milk is safe for drinking, the milk is tested and cleaned. The donated milk might be fed to the baby in a special cup, spoon, or palladai. Donor human milk and milk facilities/banks exist in other countries. Do you have any questions about this?

Question 11: Based on what I just described, how would you feel about having a donor human milk facility in your country? What are your reasons for that?

a. In your opinion, who should be responsible for establishing human milk facilities? Why?

Question 12: In general, what do you think would be needed in order to establish a donor human milk bank?

Probes

1. *Would new policies have to be put in place? If yes: What kinds of policies?*
2. *Would new safety measures have to be put in place? If yes: What kinds of measures?*
3. *Do you think it would need to be integrated with another breastfeeding promotion program? If yes: what kind of program should it be integrated with?*

Question 13: What do you think could be difficult when establishing a human milk facility?

a. What could be done to address these challenges?

Question 14: Tell me what you think would help establish a donor human milk facility**.**

SECTION IV: Prior IYCF strategies and lessons learned

Question 15: What advice do you have for people developing or testing a new intervention for low birthweight babies?

Lastly, I'd like to discuss any current or prior studies or programs that you've participated in or know about related to infant and young child feeding.

Question 16: Have you been involved with infant and young child feeding studies or programs in the past?

a. What were the outcomes?
b. (If it was successful) What made it successful?
c. What challenges did it have?

Supplement 6. In-depth interview guide for donor human milk bank experts

DONOR HUMAN MILK BANK EXPERT IDI

**SECTION I: Logistics of creating new donor human milk banks**

I'd like to start the conversation by hearing about your experience with establishing a donor human milk bank.

Question 1: Why did you decide to establish a donor human milk bank at your facility?

a. How did you decide to use human milk banks instead of using another infant feeding solution?

Question 2: At the health facility, who absolutely needed to be involved in the decision-making process?

a. How did you let people know that you were going to open a donor human milk bank? Did you use different ways for letting clinicians, staff, and moms know? What were they?
b. Were there any requirements for the facility to be eligible to have a human milk bank?

Question 3: In general, what was needed in order to implement a human milk bank from:

a. A logistical standpoint?
b. A funding standpoint?
c. A staffing standpoint?

Question 4: In general, tell me about the process after moms express and donate their milk.

a. Is their milk tested? How?
b. Is their milk cleaned/sterilized? How?

**SECTION II: Acceptability of donor human milk banks**

I'd now like to hear about the acceptability of donor human milk banks.

Question 5: Thinking of when you first introduced milk banks in your facility, please explain how women or the community felt about them.

Potential probes

Probes if not answered through question

1. *Who was the strongest supporter of human milk banks?*
2. *Where did you experience the most resistance to human milk banks?*
3. *How did you handle these challenges/resistors?*

Question 6: What information did you find useful when explaining what a human milk bank was to moms?

a. To a mom's family members?
b. To clinicians?

Question 7: Have women in your community been willing to donate their breast milk for other babies who are not able to drink their mom's own milk? Why or why not?

a. How often do women donate breast milk?
b. Are moms who donate breast milk for babies given any incentives? Why or why not?
    a. If incentivized: What do moms receive?
    b. If incentivized: Have you had any problems with giving incentives? Have there been any downsides to giving incentives?

c. If not incentivized: How did/do you get moms to donate their breast milk?

Section III: Facilitators and barriers

Thank you for all that you have shared so far. Now, I'd like to hear about the facilitators and challenges you faced when establishing a human milk bank.

Question 8: Tell me about any challenges that you faced when setting up a donor human milk bank.

a. How did you manage this challenge?

Potential probes

Probes if not answered through question

1. *Were there any cultural challenges? Religious? Health concerns for mom or baby?*
2. *Was funding a challenge when establishing the human milk bank?*
3. *Did you face any difficulties finding the tools and space to safely process and test human milk?*
4. *Was staffing a challenge when establishing a human milk bank? Did milk banks use existing health facility staff or hire their own staff members?*

Question 9: Tell me about what helped when setting up or creating a donor human milk bank.

Question 10: Describe any challenges you faced after the milk bank was up and running?

Potential probes

Probes if not answered through question

1. *Did you face any challenges with having consistent electricity for refrigeration?*
2. *Are there any current funding challenges with maintaining the milk bank?*

Question 11: Who is able to access and use human milk banks? In other words, can any mom and baby in the facility access and use the human milk bank?

Question 12: Did you find any health benefits for mom or baby after creating a milk bank in your facility? If yes: Please tell be about the benefits.

Section IV: Scaling up and sustaining a donor human milk bank program

Question 13: I'd like to get your opinion on the logistics of expanding a donor human milk program into communities and what that would look like. Thinking of your experience, what do you think would be needed to deliver donor human milk to the community or mothers who do not use your facility?

a. What do you think would help a community-based donor human milk program be successful?
b. What are some challenges that you could anticipate?

Potential probes

Probes if not answered through question

1. *What kind of delivery logistics do we need to consider? What would we need to know about the cold chain?*
2. *What kind of cost considerations do we need to consider?*
3. *In your opinion, what sort of community education would we have to rollout?*

[Question below has two options: the first for milk banks that have been sustained, the second for milk banks that have not been sustained. Please ask the appropriate option based on the interviewee's response to the main part of Q14]

Question 14: Has the human milk bank been sustained past the pilot or initial implementation phase?

If yes: Please explain how you have been able to sustain your donor human milk program:

a. From a logistical standpoint (i.e. electricity, refrigeration, hygiene/cleanliness)?
b. From a staffing standpoint?
c. From a donor milk supply standpoint?
d. From a government or policy standpoint?

If no: Why do you think the donor human milk bank at the facility was not sustained?

a. If you did try to sustain it, how did you try? What did you do?
b. If you did try to sustain it, what challenges did you experience?
c. If you did not try to sustain it, please explain why.

**CLOSING**

Question 15: Is there anything we didn't talk about today that you think we should know about establishing a milk bank?

Supplement 7. In-depth interview guide for supply chain experts

SUPPLY CHAIN EXPERT IDI

**SECTION I: How to consistently obtain and distribute sufficient quantities of each infant feeding strategy**

We would like to test different infant feeding strategies so that we can create recommendations for governments, clinicians and parents to follow when low birthweight infants are not growing and gaining weight similar to other infants of their age. One method is to have women feed their low birthweight baby formula or supplements.

Question 1: In general, please explain the process for distributing the following in your country:

[Only ask about what is applicable to the interviewee and their expertise/role]

a. Vitamins or minerals for babies
b. Infant feeding supplies
c. Bottled or treated water
d. Infant formula

**SECTION II: Barriers and facilitators to a reliable supply chain**

I'd like to hear about your experience with maintaining a reliable supply chain. I am going to ask you questions about how often you distribute certain items as well as any challenges or facilitators that you may face.

Question 2: What infant feeding supplies do you most often distribute to health facilities?

Potential probes

Probe if not answered through question

1. *Do you distribute bottled or treated water? Is there a certain brand that you distribute?*
2. *Do you distribute formula? Is there a certain brand that you distribute?*
3. *Do you distribute bottles? Is there a certain brand that you distribute?*
4. *Do you distribute vitamins or supplements? Is there a certain brand that you distribute?*

Question 3: In general, how often do you distribute baby formula or breast milk substitutes to a health facility?

Potential probes

Probe if not answered through question

1. *Are you able to consistently access different types of formula? For example, preterm vs. standard vs. human milk fortifier? What about different brands of formula?*

Question 4: In general, how often do you distribute treated or bottled water to a health facility?

Question 5: Tell me about any problems that you have experienced when supplying [BABY FORMULA/TREATED OR BOTTLED WATER/SUPPLEMENTS] to facilities.

a. How often does that happen?
b. What have you done when that has happened?
c. How long does it typically take to return to normal distribution?
d. Where do you often see the biggest breakdown in the supply chain when distributing [BABY FORMULA/TREATED OR BOTTLED WATER/SUPPLEMENTS]?

Potential probes

Probe if not answered through question

1. *Tell me about any problems with the roads that you may encounter when supplying infant feeding materials to facilities.*
2. *Describe any seasonal or weather-related challenges you may face when supplying infant feeding materials to facilities.*
3. *Is fuel scarcity ever an issue when supplying resources to facilities?*
4. *Tell me about any political challenges you have faced when trying to supply these resources to facilities.*

Question 6: It sounds like there are some challenges that you have encountered when supplying facilities with infant feeding supplies. Can you now please tell me about what helps facilitate the distribution of [BABY FORMULA/TREATED OR BOTTLED WATER]?

Question 7: Thank you for all that you have shared so far. Thinking ahead, we'd like to create an infant feeding project that might require using baby formula, treated water, and vitamins and minerals. These supplies will be needed in large, consistent quantities. In your opinion, what would be needed to ensure timely delivery of these different infant feeding supplies?

CLOSING

Question 8: Is there anything else about receiving and distributing infant feeding products that we did not discuss today that you think we should know?
